# METTL1-mediated m^7^G modification regulates hair follicle cycle via the HOXC13/FOXN1/DSG4 axis

**DOI:** 10.1371/journal.pgen.1012307

**Published:** 2026-09-22

**Authors:** Xinyan Gan, Qiwen Li, Qiuchan Xiong, Denghao Huang, Zizheng Liu, Qi Yin, Shuang Jiang, Takuma Matsubara, Shoichiro Kokabu, Wei Yan, Quan Yuan

**Affiliations:** 1 State Key Laboratory of Oral Diseases & National Center for Stomatology & National Clinical Research Center for Oral Diseases, West China Hospital of Stomatology, Sichuan University, Chengdu, Sichuan, China; 2 Department of Oral Implantology, West China Hospital of Stomatology, Sichuan University, Chengdu, Sichuan, China; 3 West China School of Medicine, Sichuan University, Sichuan University affiliated Chengdu Second People's Hospital, Chengdu, Sichuan, China; 4 Division of Molecular Signaling and Biochemistry, Kyushu Dental University, Kitakyushu, Fukuoka, Japan; 5 Department of Dermatology and Venereology, West China Hospital, Sichuan University, Chengdu, Sichuan, China; University of Oxford, UNITED KINGDOM OF GREAT BRITAIN AND NORTHERN IRELAND

## Abstract

Rapid hair follicle cycling requires precise fate commitment and differentiation of hair follicle stem cells. While the essential role of transcription factors in establishing cell identity is well recognized, how cells control its protein synthesis to achieve tissue specificity remains unknown. RNA modifications constitute a pivotal layer of post-transcriptional regulation for protein synthesis. Among them, the *N*^7^-methylguanosine (m^7^G) modification has recently emerged as a critical regulator with diverse functional impacts. Here, we reveal a pivotal role of METTL1, the key enzyme for RNA m^7^G modification, in hair follicle cycle. Conditional knockout of *Mettl1* in keratinocytes leads to severe hair follicle developmental dysplasia, impaired regeneration, and disrupted keratinocyte adhesion. Complementarily, keratinocyte-specific knock-in of *Mettl1* accelerates hair regeneration. Mechanistically, beyond its effects on tRNAs, METTL1 deficiency destabilizes *HOXC13* mRNA through internal m^7^G modification, which in turn downregulates the HOXC13/FOXN1/DSG4 signaling axis. Our findings establish METTL1-mediated internal mRNA m^7^G methylation as one of the essential regulatory layers of RNA modification in hair follicle morphogenesis and cycling, operating through the precise post-transcriptional control of a key transcriptional circuit to ensure structural integrity and timely regeneration.

## Author summary

We investigated whether METTL1, an enzyme that adds a chemical mark (m^7^G) to RNA molecules, plays a role in hair follicle growth and regeneration. Using mice in which we deleted *Mettl1* specifically in keratinocytes, we found that these animals developed abnormal hair follicles and could not regrow hair after injury. Conversely, when we increased *Mettl1* expression in keratinocytes, hair regrowth sped up. Looking at the underlying mechanism, we discovered that METTL1 stabilizes the messenger RNA of a key regulator called *HOXC13*. Without METTL1, HOXC13 levels drop, leading to reduced expression of its downstream targets FOXN1 and DSG4, which are essential for keeping hair shaft cells tightly attached to each other. As a result, keratinocyte adhesion weakens, and hair structure is compromised. Our work identifies METTL1‑mediated m^7^G modification as a critical post‑transcriptional control that ensures proper hair follicle development and timely cycling by fine‑tuning a key gene regulatory network.

## Introduction

As one of the most precise examples of cyclical tissue remodeling in mammals, the hair follicle (HF) demands the accurate and timely synthesis of a vast array of structural proteins, enzymes, and signaling molecules to rebuild the hair shaft and its surrounding layers. Upon proper molecular signals or stimuli, quiescent hair follicle stem cells (HFSCs) become activated. These activated HFSCs then orchestrate anagen onset, thereby initiating a new hair follicle cycle and initiating a differentiation program. While the morphological stages and key signaling pathways governing the hair follicle cycle and stem cell differentiation are well-described, a central question remains: how is the protein synthesis machinery itself regulated with the speed and precision required for hair follicle development and regeneration? Protein synthesis is achieved through a process called mRNA translation, which is regulated by diverse signaling. RNA modification is one of the critical regulatory layers of mRNA translation and protein synthesis [1–3]. Several studies have revealed the crucial role of RNA modification in hair follicle development and HFSCs fate decision. For example, FTO-mediated m^6^A modification of lncRNA AC010789.1 was reported to promote HFSCs growth by activating S100A8/Wnt/β-catenin signaling [4]. Furthermore, NSUN2 (NOP2/Sun RNA methyltransferase 2)-mediated tRNA m^5^C methylation regulates the development of skin and the differentiation of HFSCs via controlling protein synthesis, as its deletion leads to tRNA cleavage, accumulation of inhibitory 5′ tRNA fragments [5].

RNA modifications play crucial roles in regulating RNA metabolism and function, with broad relevance to both normal physiology and diseases. These chemical alterations directly influence RNA stability, localization, splicing and degradation, thereby fine‑tuning the accuracy and efficiency of protein synthesis [1]. Through these molecular mechanisms, RNA modification regulates key biological processes, such as stem cell fate determination, embryonic development, tumorigenesis [6–9]. As a crucial post-transcriptional modification, *N*^7^-methylguanosine (m^7^G) modification occurs at nucleotide 46 within the variable loop of certain tRNAs and at internal mRNAs, contributing to the maintenance of RNA integrity and stability [10]. Methyltransferase 1 (METTL1), along with its binding protein, WD repeat-containing protein 4 (WDR4), is the key methyltransferase responsible for tRNA and internal mRNA m^7^G modification [11]. Dysregulation of tRNA m^7^G modification caused by abnormal expression of METTL1 has been implicated in both development and diseases [12–14]. Importantly, the internal mRNA m^7^G modification levels are relatively low (approximately 0.02% to 0.05%) across several human and mouse cell lines [15], but these modifications substantially influence mRNA stability and translation under stress conditions. This regulation occurs through the selective recruitment of m^7^G-modified transcripts to stress granules (SGs) via the RNA-binding protein Quaking Protein Isoform 7 (QKI7), which binds through its C‑terminus to the SG core protein G3BP1 [16]. In recent years, several studies has reported that METTL1-mediated internal mRNA m^7^G modifications play critical roles in various biological processes, such as rheumatoid arthritis, macrophage inflammatory responses and multiple organ injury [17–19]. However, the role of METTL1-mediated m^7^G modifications in hair follicle and HFSCs function remains poorly understood.

Hair loss, associated with a pooled anxiety disorder prevalence of 47% among patients [20], is a major source of psychological distress, underscoring the importance of research on hair follicle development and regeneration as a key scientific and healthy focus [21–23]. The hair follicle undergoes life-long, repetitive cycles of regressing (catagen), resting (telogen), and growing phase (anagen) [24]. Besides, hair follicles are pivotal and multi-faceted regulators of skin barrier function [25]. While protein synthesis activity is the highest in the growing phase of hair follicle, the precise regulatory mechanisms that control this coordination remain elusive.

Therefore, in this study, we aim to investigate whether METTL1 also contributes to the regulation of hair follicle development and regeneration. By generating *Mettl1* conditional knockout and knock-in mice, we revealed that METTL1-mediated internal mRNA m^7^G modification is essential in both hair follicle development and regeneration process. This regulation primarily functions by stabilizing *HOXC13* mRNA to sustain its protein level, which in turn drives the synthesis of key downstream effector proteins. Collectively, our findings establish mRNA internal m^7^G modification as one of the critical regulatory layers that links translational control to hair follicle development and regeneration.

## Results

### METTL1 is highly expressed in mice dorsal skin and hair follicle

**Fig 1:**
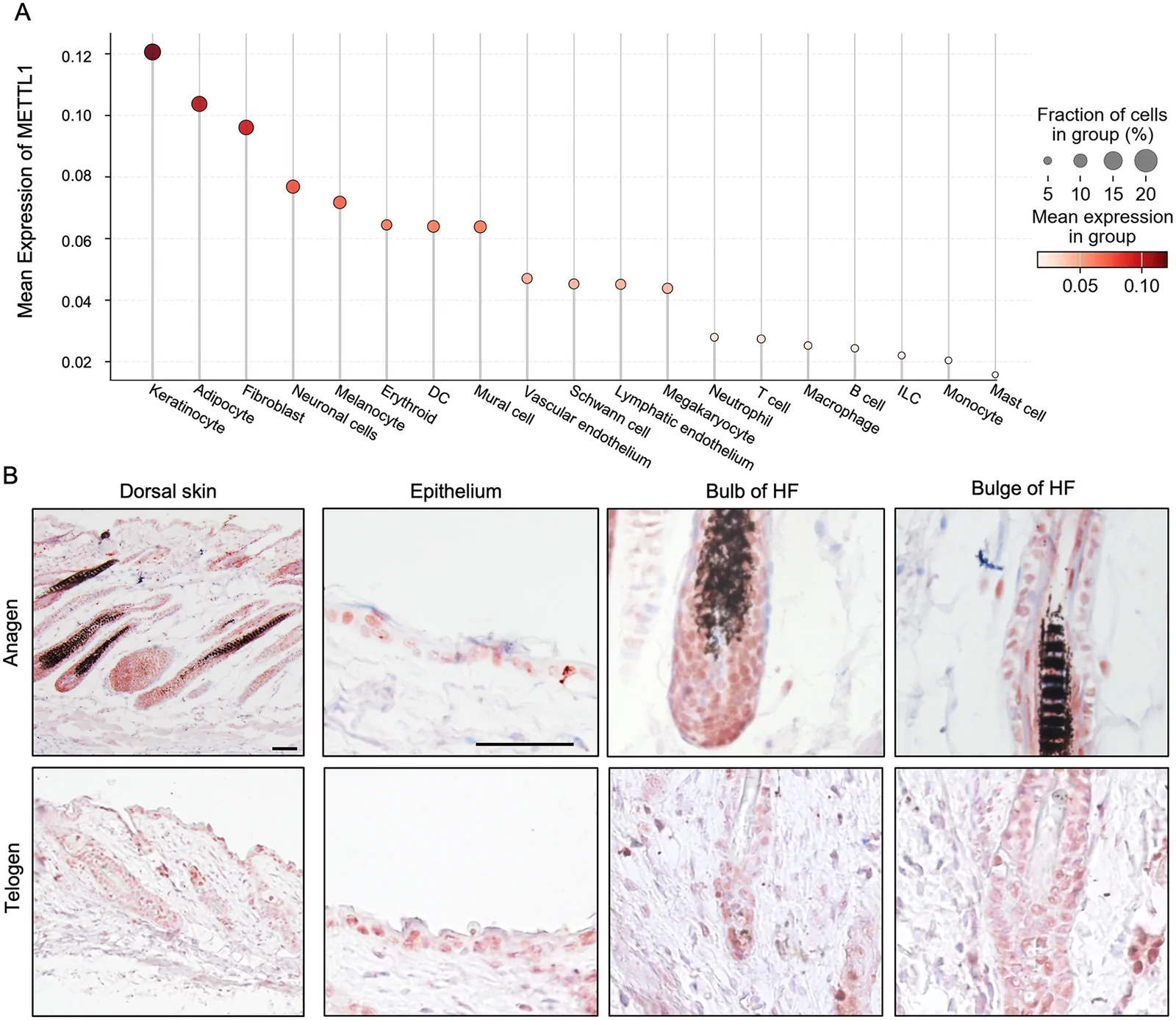
METTL1 expression in dorsal skin. A. The lollipop chart showing the expression levels of *METTL1* across major cell types identified in a single-cell RNA sequencing dataset of human embryonic skin B. Representative immunohistochemical staining images of METTL1 in anagen and telogen HFs of mice. Scale bar, 50μm.

Firstly, through analysis of a single-cell dataset of prenatal human skin (7–17 post-conception weeks) [26], we found that keratinocytes are among the cell types with the highest expression of *METTL1* (Fig 1A).To determine the expression pattern of METTL1 in dorsal skin and hair follicle of mice throughout the hair cycle, the immunohistochemical staining was performed. METTL1 was specifically expressed in basal cells of skin epithelium, and in the bulge and bulb of hair follicles both in the telogen and anagen phase (Fig 1B). These findings show that METTL1 is consistently and highly expressed in key epithelial and hair follicle compartments throughout skin development.

### Conditional knockout of *Mettl1* leads to hair development dysplasia

**Fig 2:**
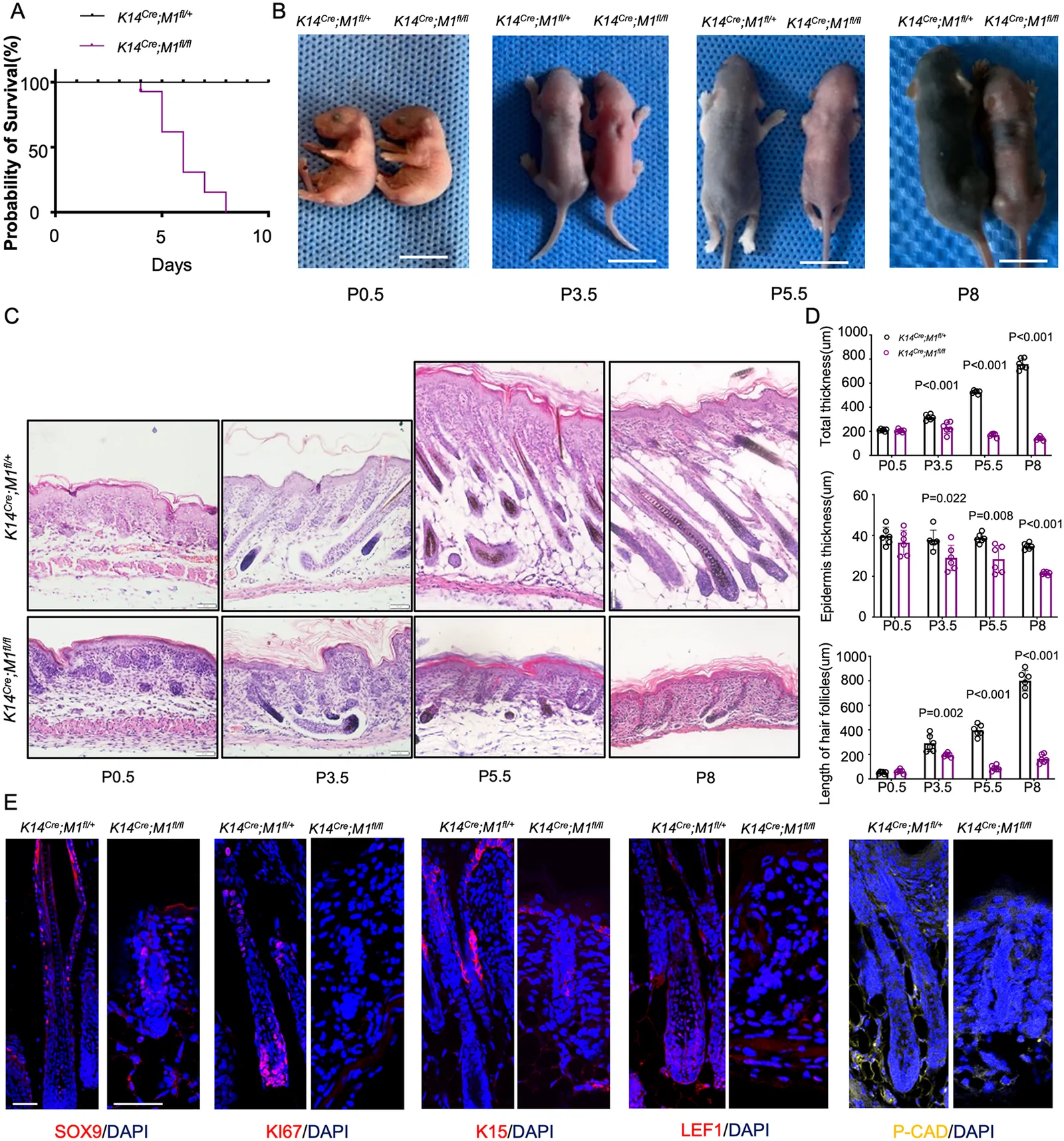
Conditional knockout of *Mettl1* leads to hair development dysplasia of mice. A. The survival curve of *K14*^*Cre*^*;Mettl1*^*fl/fl*^ and *K14*^*Cre*^*;Mettl1*^*fl/+*^ mice within the 10 days prior to birth (n = 10). B. Representative images of *K14*^*Cre*^*;Mettl1*^*fl/fl*^ and *K14*^*Cre*^*;Mettl1*^*fl/*^+ mice at P0.5, P3.5, P5.5 and P8. C. Representative HE staining images of dorsal skin of *K14*^*Cre*^*;Mettl1*^*fl/fl*^ and *K14*^*Cre*^*;Mettl1*^*fl/*^+ mice at P0.5, P3.5, P5.5 and P8. Scale bar, 50μm. D. Quantitative analysis of total thickness, epidermal thickness and the length of HFs of dorsal skin from *K14*^*Cre*^*;Mettl1*^*fl/fl*^ and *K14*^*Cre*^*;Mettl1*^*fl/*^+ mice at P0.5, P3.5, P5.5 and P8 (n = 6). E. Representative confocal images of HFs from *K14*^*Cre*^*;Mettl1*^*fl/fl*^ and *K14*^*Cre*^*;Mettl1*^*fl/+*^ mice at P5.5. Scale bar, 100μm.

To test whether METTL1-mediated m^7^G modification plays a role in hair follicle development, we generated *Mettl1* conditional knockout mice using *K14*^*Cre*^, which selectively deletes *Mettl1* in epithelial cells (*K14*^*Cre*^*;Mettl1*^*fl/fl*^). The *K14*^*Cre*^*;Mettl1*^*fl/fl*^ newborn mice survived for a maximum of only 8 days, as indicated by the survival probability curve presented in Fig 2A. Gross observation of *K14*^*Cre*^*;Mettl1*^*fl/fl*^ mice and their wild-type (WT) littermates showed that there were no visible differences in dorsal skin at Postnatal day 0.5 (P0.5). However, the *K14*^*Cre*^*;Mettl1*^*fl/fl*^ mice exhibited smaller body size and lighter pigmentation from P3.5 to P8, suggesting developmental dysplasia of skin and hair follicle (Fig 2B). H&E staining and quantification showed that the cross-sectional total thicknesses of skin in *K14*^*Cre*^*;Mettl1*^*fl/fl*^ mice were smaller than their littermates at P3.5, P5.5 and P8 (Fig 2C–2D). However, there is no visible difference in the epidermis thickness of dorsal skin between *K14*^*Cre*^*;Mettl1*^*fl/fl*^ mice and their littermates at P0.5, P3.5 and P5.5, except at P8, which is thinner (Fig 2D). Besides, we did not find visible difference of the skin barrier between *K14*^*Cre*^*;Mettl1*^*fl/fl*^ mice and littermates at E18.5 (S1 Fig). More importantly, the hair follicle of *K14*^*Cre*^*;Mettl1*^*fl/fl*^ mice displayed phenotypes characterized by notably abnormal hair shaft morphology that failed to emerge, in contrast to WT controls (Fig 2C–2D).

We further performed immunostaining to investigate the development of mouse dorsal hair follicles. We chose Keratin 15 (K15) as the maker for bulge stem cells, SRY-Box Transcription Factor 9 (SOX9) for HFSCs maintenance, Placental Cadherin (P-CAD) for hair shaft structural integrity, and Lymphoid Enhancer Binding Factor 1 (LEF1) for Wnt pathway activation [21]. Combined analysis of their expression allows for the evaluation of key processes from HFSCs preservation to differentiation. An apparent reduction in the expression level of K15, SOX9, P-CAD and LEF1 was observed across all hair follicles in *K14*^*Cre*^*;Mettl1*^*fl/fl*^ mice (Fig 2E). Besides, the number of Ki67^+^ proliferating cells was significantly reduced in hair follicles of dorsal from *K14*^*Cre*^*;Mettl1*^*fl/fl*^ mice (Fig 2E). We further tested cell apoptosis via Terminal dUTP Nick-End Labeling (TUNEL) staining, which revealed a slight increase of apoptosis in hair follicles from *K14*^*Cre*^*;Mettl1*^*fl/fl*^ mice (S2 Fig). In summary, these results demonstrate that the knockout of *Mettl1* leads to developmental impairments in mouse dorsal hair follicles.

### METTL1 is essential for hair regeneration

**Fig 3:**
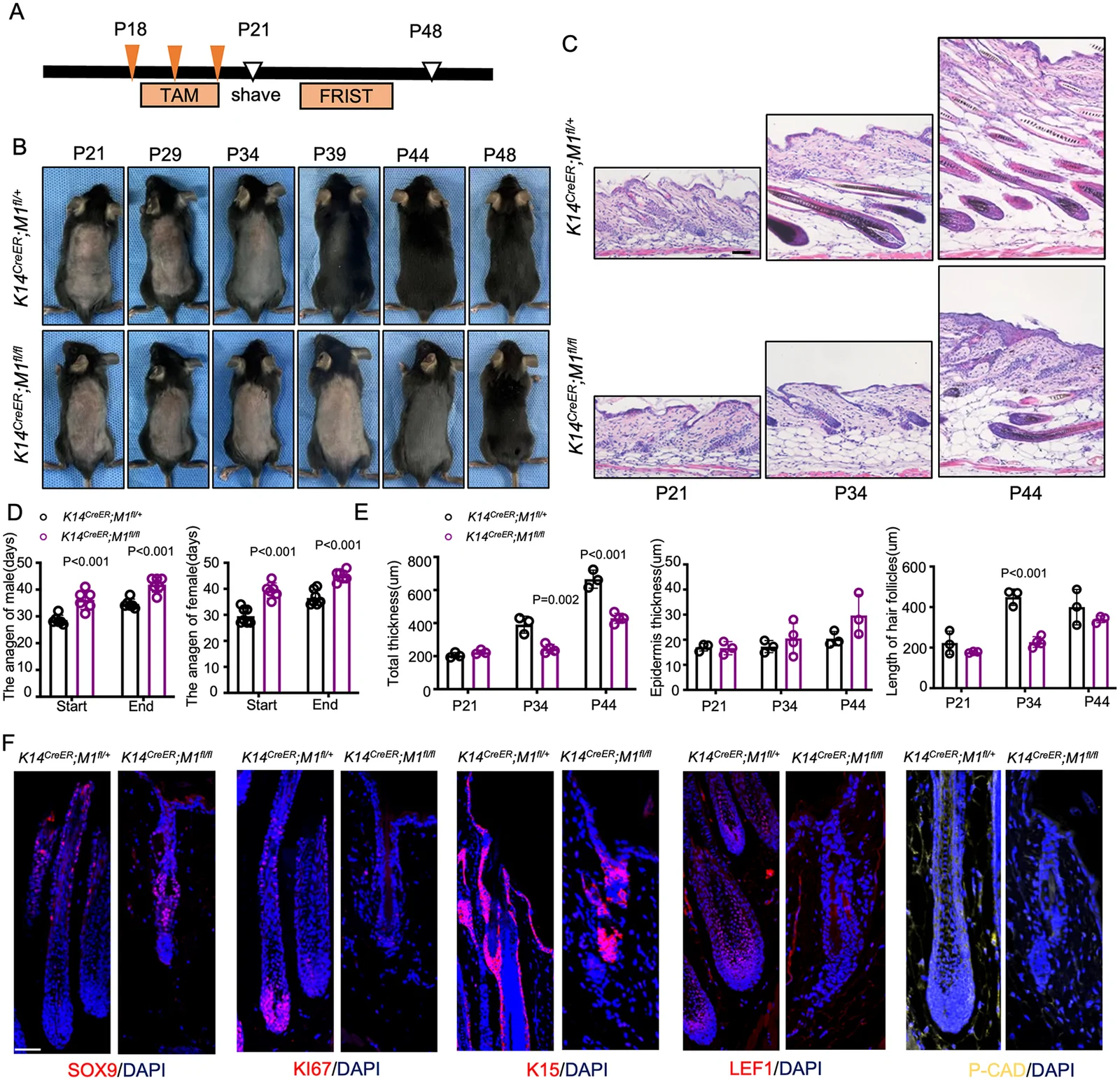
Conditional knockout of *Mettl1* delays hair regeneration. A. The flowchart of tamoxifen induction and hair-shaving treatment to *K14*^*CreER*^*;Mettl1*^*fl/fl*^ and *K14*^*CreER*^*;Mettl1*^*fl/+*^ mice. The mice were treated with tamoxifen induction at P18. The hair in dorsal skin was shaved at P21(in telogen) and observed daily for hair regeneration in both *K14*^*CreER*^*;Mettl1*^*fl/fl*^ and *K14*^*CreER*^*;Mettl1*^*fl/+*^ mice. B. Representative and detailed images of hair regeneration from *K14*^*CreER*^*;Mettl1*^*fl/fl*^ mice and *K14*^*CreER*^*;Mettl1*^*fl/*^+ mice after tamoxifen induction and hair-shaving treatment (female). C. Representative HE staining images of dorsal skin from *K14*^*CreER*^*;Mettl1*^*fl/fl*^ mice and *K14*^*CreER*^*;Mettl1*^*fl/*^+ mice after tamoxifen induction and hair-shaving treatment at P21, P34 and P44. Scale bar, 50μm. D. Quantitative analysis of the start days and done days of the hair regeneration between *K14*^*CreER*^*;Mettl1*^*fl/fl*^ and *K14*^*CreER*^*;Mettl1*^*fl/*^+ mice in male and female severally (n ≥ 6). E. Quantitative analysis of total thickness, epidermal thickness, and the length of HFs of dorsal skin from *K14*^*CreER*^*;Mettl1*^*fl/fl*^ and *K14*^*CreER*^*;Mettl1*^*fl/*^+ mice at P21, P34 and P44 (n ≥ 3). F. Representative confocal images of the HFs from *K14*^*CreER*^*;Mettl1*^*fl/fl*^ mice and *K14*^*CreER*^*;Mettl1*^*fl/+*^ mice after tamoxifen induction and hair-shaving treatment at P34. Scale bar, 100μm.

Because of the early lethality of *K14*^*Cre*^*;Mettl1*^*fl/fl*^ newborn mice, we further generated the tamoxifen-induced *K14*^*CreER*^*;Mettl1*^*fl/fl*^ mice. The IHC staining of METTL1 demonstrated efficient knockout (S3A Fig). The first hair follicle regeneration cycle was observed after 3 days of tamoxifen injection and subsequent hair shaving (Fig 3A), as previously described [21]. Gross observation showed a delay in whole hair follicle cycle of *K14*^*CreER*^*;Mettl1*^*fl/fl*^ mice, compared with their littermates controls (Figs 3B, S3B). H&E staining and quantifications revealed that at P21, hair follicles in both *K14*^*CreER*^*;Mettl1*^*fl/fl*^ mice and littermate controls were in the telogen phase. By P34, whereas the *K14*^*CreER*^*;Mettl1*^*fl/+*^ hair follicles had entered the anagen phase, *K14*^*CreER*^*;Mettl1*^*fl/fl*^ hair follicles remained arrested in telogen phase, which lasts until around P44, both in male and in female mice (Fig 3C–3E). The immunostaining of K15, SOX9, P-CAD, LEF1 and Ki67 revealed that the hair follicle of *K14*^*CreER*^*;Mettl1*^*fl/fl*^ mice at P34 remains at the telogen stage while that of control mice proceeds to anagen stage (Fig 3F). The above results demonstrate that METTL1 is essential for the timely initiation of hair follicle regeneration, with its loss leads to a prolonged hair follicle renewal time in mice.

### METTL1 regulates the hair follicle integrity and regeneration by HOXC13/FOXN1/DSG4 axis

**Fig 4:**
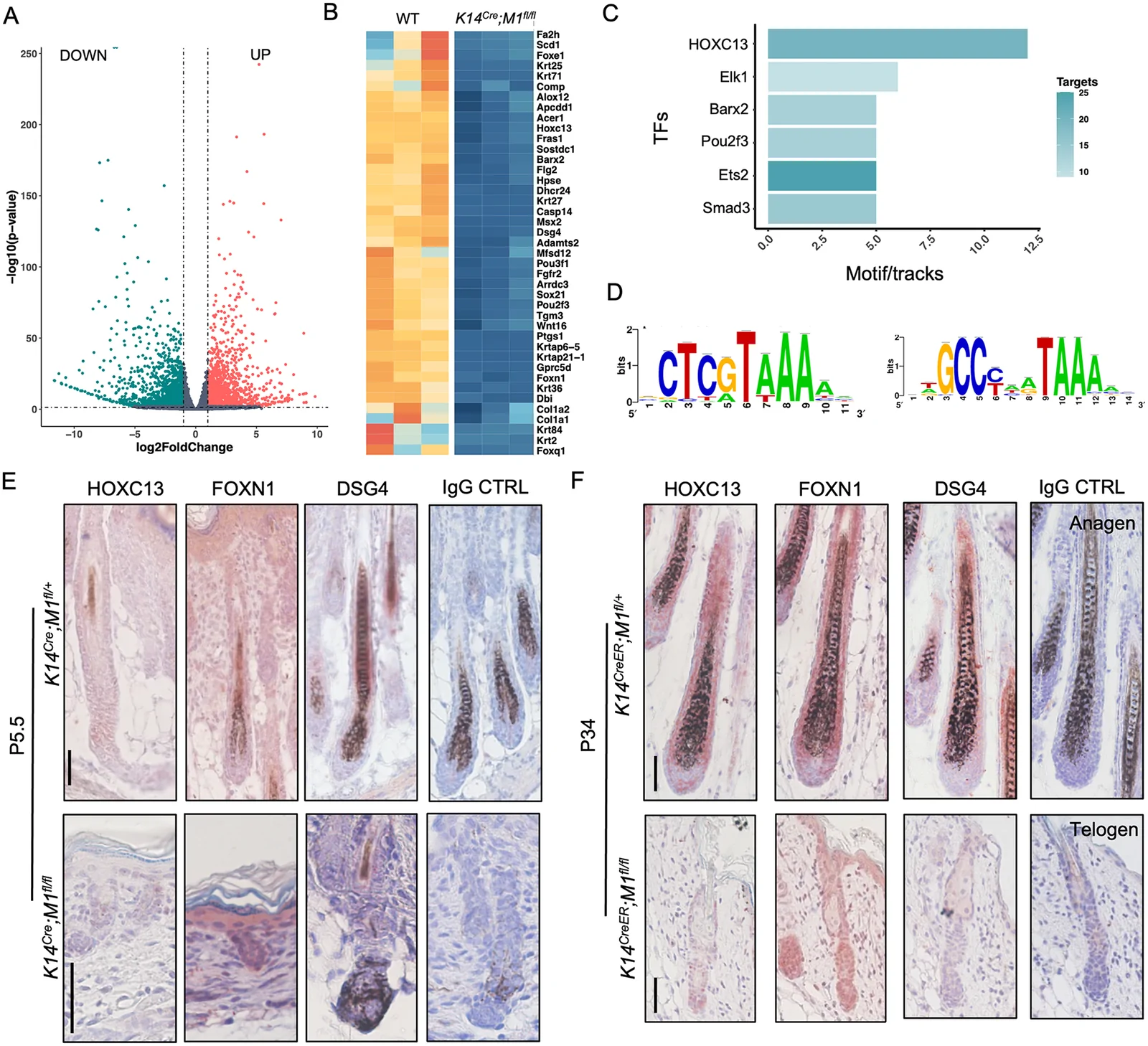
METTL1 regulates the HF integrity and regeneration by HOXC13/FOXN1/DSG4 axis. A. Volcano plot showing the differentially expressed genes. B. The heatmap of differentially expressed genes related to the skin development. C. The bar plot showing the transcription factors most likely to regulate the common characteristics of these 41 downregulated genes identified through enrichment analysis. D. The two motifs most frequently regulated by HOXC13. E. Representative immunohistochemical staining images of HFs from *K14*^*Cre*^*;Mettl1*^*fl/fl*^ and *K14*^*Cre*^*;Mettl1*^*fl/*^+ mice at P5.5. Scale bar, 100μm. F. Representative immunohistochemical staining images of the HFs from *K14*^*CreER*^*;Mettl1*^*fl/fl*^ mice and *K14*^*CreER*^*;Mettl1*^*fl/+*^ mice after tamoxifen induction and hair-shaving treatment at P34. Scale bar, 100μm.

To reveal the signaling impaired after *Mettl1* knockout, we performed RNA sequencing (RNA-seq) of the epithelium of dorsal skin from the *K14*^*Cre*^*;Mettl1*^*fl/fl*^ mice and their littermates at P5. We have identified a total number of 2997 genes differentially expressed, with 1459 upregulated and 1538 downregulated. Volcano plot showing the differentially expressed genes (Fig 4A). We further sorted genes relating to skin development and the heatmap showed 41 differentially expressed genes after *Mettl1* knockout (Fig 4B). As METTL1 functions primarily at the translational level, we speculate that these differentially expressed genes may result from impaired translation of upstream regulatory factors. Therefore, we further performed transcription enrichment analysis to predict potential transcription factors that leads to this differential expression. We identified that the transcription factor Homeobox C13 (HOXC13) is the most enriched transcriptional factor that regulates half of the differentially expressed skin development-related genes (Fig 4C). These genes have a characteristic TAA sequence core motif that is bound by HOXC13 [27] (Fig 4D). HOXC13 acts as a central regulator orchestrating the expression of intermediate filament proteins, known as hair keratins [28,29]. Loss-of-function mutations in *HOXC13* cause ectodermal dysplasia-9 (ED-9) in humans, characterized by hypotrichosis and nail dystrophy [30]. Notably, *Hoxc13*^*tm1Mrc*^ mice exhibited a nude phenotype due to malformed hair shafts [31], closely resembling the phenotype observed in *K14*^*Cre*^*;Mettl1*^*fl/fl*^ mice.

Based on this phenotype similarity, we hypothesize that reduced HOXC13 protein level might contribute to hair follicle defect observed in *K14*^*Cre*^*;Mettl1*^*fl/fl*^ mice phenotype. To test this hypothesis, we examined HOXC13 expression and downstream regulatory network. HOXC13 has been reported to regulate hair follicle development and regeneration through the Forkhead Box N1 (FOXN1)-Desmoglein 4 (DSG4) axis [31,32]. Consistent with this model, RNA-seq analysis revealed a significant downregulation of *Foxn1* and *Dsg4* in *K14*^*Cre*^*;Mettl1*^*fl/fl*^ mice (Fig 4B). We further performed immunohistochemical staining and the results showed that the protein level of HOXC13, FOXN1 and DSG4 was markedly reduced in hair follicle of *K14*^*Cre*^*;Mettl1*^*fl/fl*^ mice, compared with their littermate controls at P5 (Fig 4E). Moreover, reduced expression of HOXC13, FOXN1 and DSG4 was also observed in the telogen hair follicle of *K14*^*CreER*^*;Mettl1*^*fl/fl*^ mice, in contrasted to control mice that already entered the subsequent anagen phase in the regeneration model (Fig 4F).

### Depletion of *Mettl1* impairs the intercellular adhesion of keratinocytes

**Fig 5:**
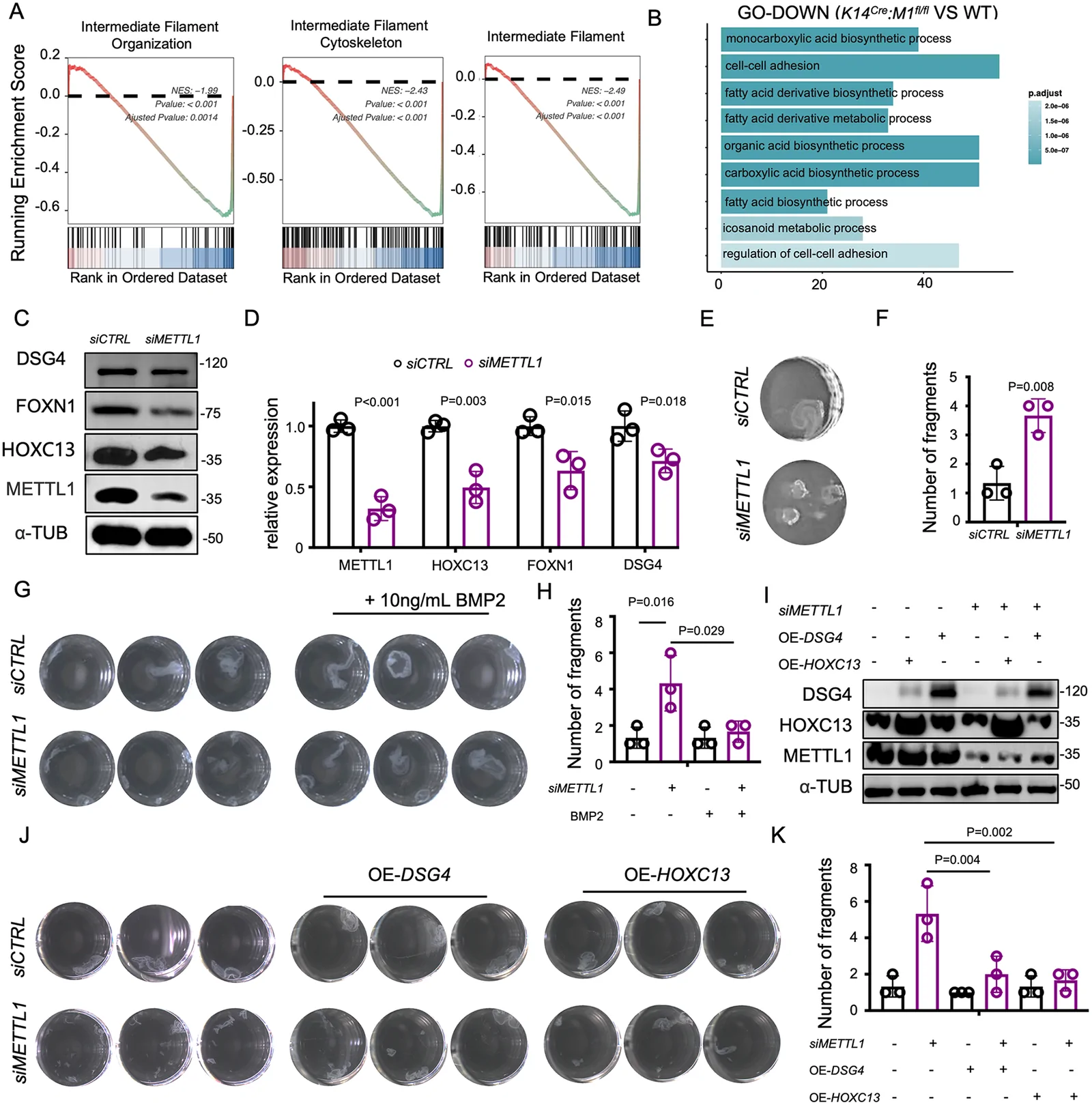
Depletion of *Mettl1* impairs the intercellular adhesion of Keratinocytes. A. Gene sets enrichment analysis of the down-regulated pathways. B. GO enrichment analysis of the down-regulated pathways. C-D. Representative Western blot images and quantitative analyses of METTL1, DSG4, FOXN1 and HOXC13 in *siCTRL* and *siMETTL1* HaCaTs (n = 3). E-F. Representative images and quantitative analyses of Dispase-based dissociation assays in *siCTRL* or *siMETTL1* HaCaTs (n = 3). G-H. Representative images and quantitative analyses of Dispase-based dissociation assays in *siCTRL* or *siMETTL1* HaCaTs, partially treated with BMP2 (n = 3). Each dot represents one biological replicate. I. Representative Western blot images of METTL1, DSG4 and HOXC13. J-K. Representative images and quantitative analyses of Dispase-based dissociation assays in *siCTRL* or *siMETTL1* HaCaTs (n = 3), partially overexpressed *DSG4 or HOXC13*. Each dot represents one biological replicate.

Given the impairment of hair follicle during development and regeneration in *K14*^*Cre*^*;Mettl1*^*fl/fl*^ mice, we examined if METTL1 depletion affected keratinocytes, the major cells responsible for hair follicle development and cyclic regeneration. We analyzed the RNA-seq of the epithelium of dorsal skin from the *K14*^*Cre*^*;Mettl1*^*fl/fl*^ mice and their littermates using gene sets enrichment analysis (GSEA) and GO analysis. GSEA revealed the most down-regulated pathways were associated with the intermediate filament organization, a process essential for hair shaft formation, as the hair shaft is largely composed of keratin intermediate filaments remaining after the programmed cell death of hair shaft keratinocytes (Fig 5A). In parallel, GO analysis showed that cell-cell adhesion-related processes were significantly impaired upon *Mettl1* deletion (Fig 5B). Proper intercellular adhesion is indispensable for hair shaft integrity, as it allows hair shaft cells to function as a cohesive unit, and together with intermediate filament, enables the hair fiber to withstand mechanical stress [33,34]. In hair shaft, intercellular adhesion is primarily mediated by desmosomes, specialized junctional complexes that tightly connect keratinocytes through intermediate filaments. Among desmosomal components, DSG4 is the major cadherin specifically enriched in hair shaft desmosomes and is critical for hair shaft differentiation and mechanical strength [32]. Mutations in *DSG4* disrupt desmosomal adhesion, leading to hair fragility and loss, underscoring its essential role in building and maintaining hair fiber integrity [35,36].

We thus examined the role of METTL1 in regulating keratinocyte intercellular adhesion *in vitro* using HaCaTs, an immortalized human keratinocyte line widely used to model epidermal adhesion. This system allowed us to determine whether the defects observed *in vivo* reflect a cell-autonomous function of METTL1 in keratinocytes. siRNA‑mediated knockdown of *METTL1* in HaCaTs resulted in reductions in the protein levels of HOXC13, FOXN1, and DSG4 (Fig 5C–5D). Functionally, the cell adhesion was slightly inhibited after *METTL1* depletion, as shown by CCK-8 assays (S4A Fig). More importantly, *METTL1* depletion led to impaired intercellular adhesion of HaCaTs, as shown by dispase-based dissociation assays (Fig 5E–5F).

Given the essential role of DSG4 in mediating the intercellular adhesion of keratinocytes and the fact that BMP ligand BMP2 is a potent inducer of endogenous *Dsg4* expression in mice [37], we next examined whether enhancing DSG4 expression could rescue the intercellular adhesion defect caused by *METTL1* depletion. Indeed, *METTL1-*depleted keratinocytes readily dissociated into multiple fragments upon Dispase II and mechanical stress, indicative of compromised intercellular adhesion. Notably, BMP2 treatment substantially restored cell cohesion and reduced fragmentation (Fig 5G–5H). To further rule out the possibility that BMP2 exerts this effect through activation of other downstream targets, we directly overexpressed *DSG4 or HOXC13* in *METTL1*-depleted HaCaTs using a lentiviral system. Western blot analysis confirmed stable overexpression of *DSG4* or *HOXC13* under *METTL1*-deficient cells (Figs 5I, S4B). Moreover, increased expression of *HOXC13* partially rescued DSG4 protein levels reduced by *METTL1* knockdown. Similar to the results observed in BMP2-treated cells, overexpression of *HOXC13* or *DSG4* was sufficient to rescue the intercellular adhesion defect of *METTL1*-depleted keratinocytes (Fig 5J–5K), demonstrating that impaired HOXC13 and DSG4 expression is a major contributor to the adhesion phenotype caused by METTL1 loss. Together, these results further support the functional relevance of the HOXC13-FOXN1-DSG4 axis downstream of METTL1 in regulating keratinocyte intercellular adhesion.

### METTL1 mediates internal mRNA m^7^G modification of *HOXC13*

**Fig 6:**
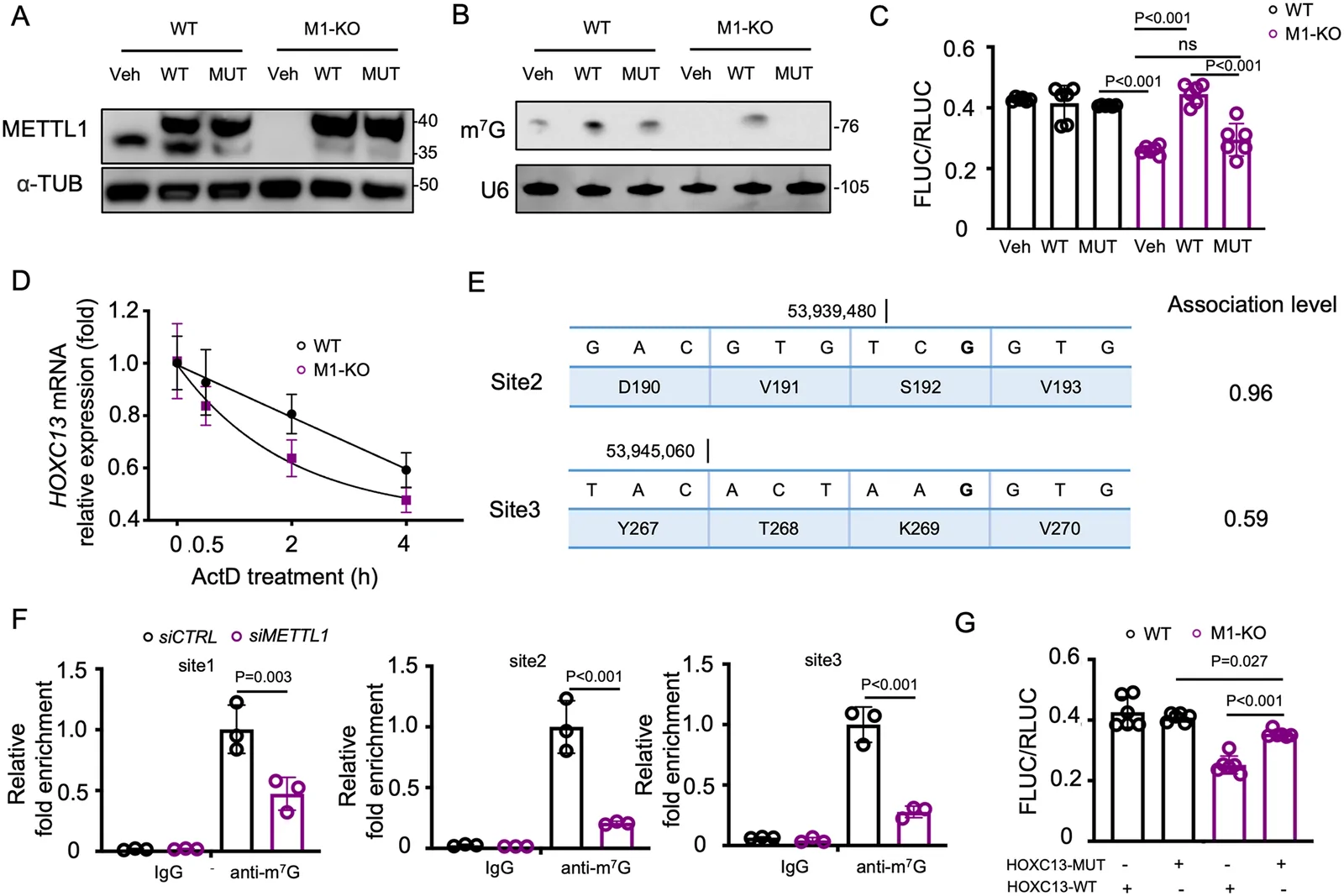
METTL1 mediates internal mRNA m^7^G modification of *HOXC13.* A-B. Representative Western blot, Northern blot and Northwestern blot images in WT and *METTL1*-KO 293Ts with the expression of the Veh, WT-METTL1 and mutant-METTL1. C. Bar plot of dual-luciferase assay in WT and *METTL1*-KO 293Ts with the expression of the Veh, WT-METTL1 and mutant-METTL1 (n = 6). D. Dot plot of mRNA stability assay in WT and *METTL1*-KO 293Ts (n = 3). E. The positions and association level of the two predicted internal m⁷G sites within the HOXC13 CDS. F. Bar plot of m^7^G MeRIP assay in *siCTRL* and *siMETTL1* HaCaTs (n = 3). G. Bar plot of dual-luciferase assay in WT and *METTL1*-KO 293Ts using dual‑luciferase reporters with/without point mutations in the two predicted m^7^G sites (n = 6).

We next investigated into the mechanism by which *METTL1* deletion reduced HOXC13 level. We first performed the dual-luciferase assay in HEK 293T cells to evaluate the effect of *METTL1* depletion on *HOXC13* mRNA translation. *METTL1* knockdown significantly impaired the translation of *HOXC13* mRNA, as evidenced by a decrease in the FLUC/RLUC ratio (S5A Fig). To test if the translation of *HOXC13* relies on the m^7^G catalytic activity of METTL1, we then constructed a catalytically inactive METTL1 mutant by introducing amino acid substitutions at its enzymatic active site (L160A/D163A) and transfected it into WT and *METTL1*-knockout HEK 293T cells (S5B Fig). Despite rescue of the overall METTL1 protein level in *METTL1*-knockout cells, overexpression of the catalytically inactive METTL1 barely restored m^7^G modification level (Figs 6A–6B, S5C–S5D). The dual-luciferase assay showed that overexpression of the catalytically inactive METTL1 protein failed to rescue the mRNA translation of *HOXC13* (Fig 6C). This indicates that the regulation of HOXC13 by METTL1 is dependent on its m^7^G methyltransferase activity.

As METTL1 is best known as a tRNA m^7^G transferase, we next examined if *METTL1* deletion impairs *HOXC13* translation by selectively reducing the availability of m^7^G-modified tRNAs that decodes *HOXC13* codons. To address this possibility, we analyzed the frequency of codons decoded by m^7^G-modified tRNAs in *HOX13* relative to control gene sets. However, no significant difference was found between *HOX13* genes and control gene sets (S5E Fig). This result suggests that impaired *HOXC13* translation is unlikely to be attributed to reduced tRNA m^7^G modification. Given that METTL1 also catalyzes internal m^7^G modification on mRNAs, we firstly analyzed the publicly available m^7^G‑ methylated RNA immunoprecipitation (MeRIP)‑seq data and found clear internal m^7^G signal (site 1) within the CDS region of *HOXC13* (S5 Table) [16]. Based on the established role of METTL1-mediated internal m^7^G modification in controlling mRNA stability, we performed the mRNA stability assay to examine this regulation^16^. Indeed, *METTL1* knockout reduced the stability of *HOXC13* mRNA (Fig 6D). Beyond the internal m^7^G signals detected in the public dataset, we performed in silico prediction using the m^7^G Hub database, which identified two additional high‑confidence sites on *HOXC13* mRNA (scores 0.96 and 0.59; threshold >0.5, max = 1) (Fig 6E). We subsequently performed MeRIP‑qPCR with an m^7^G antibody on the three candidate sites (one from public data, two from prediction). Enrichment of *HOXC13* mRNA was significantly reduced at all three sites in *siMETTL1* HaCaTs compared with control cells (Fig 6F). To examine the functional dependence of *HOXC13* translation on m^7^G modification, we further constructed dual‑luciferase reporters with synonymous point mutations in the two predicted m^7^G sites. *METTL1* knockdown reduced the FLUC/RLUC ratio of the wild‑type construct, whereas the mutation of these residues partially restored this ratio, suggesting that these sites are functionally important for *METTL1*‑dependent translational control of *HOXC13* (Fig 6G).

Altogether, these results demonstrate that METTL1 sustains *HOXC13* expression level by stabilizing its transcripts through internal mRNA m^7^G modification.

### Conditional knock-in of *Mettl1* promotes the hair regeneration

**Fig 7:**
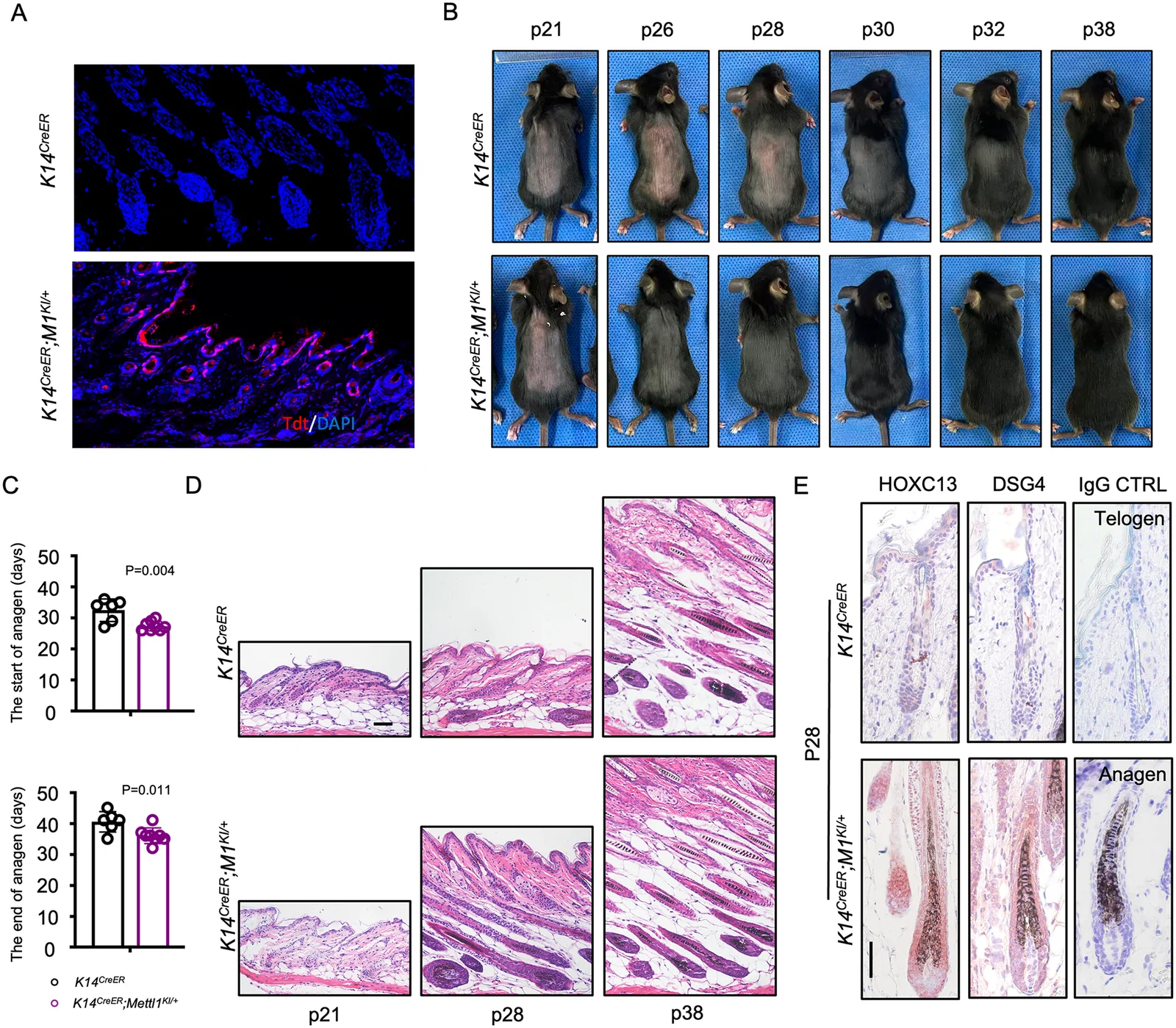
Conditional knock-in of *Mettl1* promoted hair regeneration. A. Representative images of IF staining of *K14*^*CreER*^*;Mettl1*^*KI/+*^ mice. B. Representative and detailed images of hair regeneration from wild type mice and *K14*^*CreER*^*;Mettl1*^*KI/*^+ mice after tamoxifen induction and hair-shaving treatment (female). C. Quantitative analysis of the start days and done days of the hair regeneration between wild type mice and *K14*^*CreER*^*;Mettl1*^*KI/*^+ mice after tamoxifen induction and hair-shaving treatment (n = 6). D. Representative HE staining images of dorsal skin from wild type mice and *K14*^*CreER*^*;Mettl1*^*KI/*^+ mice after tamoxifen induction and hair-shaving treatment at P21, P28 and P38. E. Representative IHC staining images of dorsal skin from wild type mice and *K14*^*CreER*^*;Mettl1*^*KI/+*^ mice after tamoxifen induction and hair-shaving treatment at P28.

The phenotype of *K14*^*CreER*^*;Mettl1*^*fl/fl*^ mice established that METTL1 is essential for hair follicle regeneration. To test whether increased METTL1 expression is sufficient to promote hair follicle regeneration, and to rule out potential indirect effects from developmental compensation in knockout models, we generated the *Mettl1* conditional knock-in mice (*K14*^*CreER*^*;Mettl1*^*KI/+*^). Immunofluorescence staining of dorsal skin confirmed that METTL1 is specifically expressed in the skin epithelium and hair follicles of *K14*^*CreER*^*;Mettl1*^*KI/+*^ mice (Fig 7A).

We next performed hair follicle regeneration experiment as described previously (Fig 3A). Compared with control mice, *K14*^*CreER*^*;Mettl1*^*KI/+*^ mice exhibited accelerated hair follicle regeneration (Fig 7B–7C). Macroscopic observation at P28 showed fully regenerated dorsal hair in *K14*^*CreER*^*;Mettl1*^*KI/+*^ mice, whereas the dorsal skin in WT littermates remained largely bald (Fig 7B). H&E staining revealed that hair follicles in *K14*^*CreER*^*;Mettl1*^*KI/+*^ mice had already entered the anagen phase by P28, while follicles in control littermates remained in telogen (Fig 7D). Immunohistochemical staining further showed increased expression of HOXC13 and DSG4 in *K14*^*CreER*^*;Mettl1*^*KI/+*^ mice (Fig 7E), suggesting that accelerated hair follicle regeneration is associated with upregulation of these key downstream effectors. Complementing the knockout phenotype, conditional knock-in of *Mettl1* in keratinocytes accelerates hair follicle renewal, demonstrating that METTL1 is both necessary and sufficient to promote the hair follicle regeneration.

## Discussion

Our study established METTL1-mediated internal mRNA m^7^G methylation as a critical regulator of hair follicle development and regeneration. By combining conditional knockout and knock-in mice models with in vitro analyses, we demonstrated that METTL1 is indispensable for both hair follicle development and regeneration. We identify a key molecular pathway whereby METTL1 deficiency disrupts the expression of a transcriptional circuit involving HOXC13 and FOXN1, leading to a downregulation of the desmosomal cadherin DSG4. The functional consequence of this disruption is a significant impairment in keratinocyte intercellular adhesion, a process fundamental to the structural integrity of the hair shaft. Crucially, we provided evidence that METTL1 regulates this axis by maintaining the mRNA stability of the central transcription factor HOXC13 through internal mRNA m^7^G modification mechanism.

Currently, the hair follicle phenotype of *K14*^*Cre*^*;Mettl1*^*fl/fl*^ mice is similar to that of *Hoxc13*^*tm1Mrc*^ mice and *Foxn1*^*nu*^ mice [31]. Although the phenotype of *Dsg4* knockout mice has not been reported, overexpression of the enzyme TghKLK14, which cleaves and degrades DSG4, also results in hair growth arrest and severe defects characterized by hyperplastic hair follicles in mice [38]. These findings suggest the critical role of HOXC13/FOXN1/DSG4 axis in hair follicles, and positions METTL1 as an upstream epi-transcriptomic regulator that sustains this axis to ensure proper hair follicle development and regeneration.

RNA modifications play crucial roles in hair follicle development and stem cell regulation, as exemplified by FTO-mediated m^6^A modification promoting HFSCs growth through Wnt/β-catenin signaling and NSUN2-dependent tRNA m^5^C methylation regulating protein synthesis during hair follicle development. However, the role of RNA modifications in hair follicle cycling is not fully understood. Beyond these known mechanisms, our work specifically underscores the importance of METTL1-mediated internal mRNA modifications in this context. Our data extended the functional repertoire of METTL1 beyond tRNA regulation to include internal mRNA m^7^G modification, a rarer but potentially impactful form of post-transcriptional control that contributes to mRNA stability. HOXC13 is a well-established master regulator of hair keratin and keratin-associated protein genes [28], and its mutation causes human ectodermal dysplasia. By demonstrating that HOXC13 functions in a METTL1-dependent pathway, we uncovered a new layer of regulation mediated by RNA modifications that controls its expression. This regulation extends to the downstream known targets FOXN1 and DSG4 [32], thereby linking an RNA modification-based regulator with a key effector of intercellular adhesion in the hair follicle.

The functional deficit in *METTL1*-deficient keratinocytes manifests primarily as an intercellular adhesion deficit. This aligns perfectly with the known biology of DSG4, a key structural and functional component of desmosomal adhesion [39]. Our rescue experiments, where both BMP2 treatment and *DSG4/HOXC13* overexpression restored the intercellular adhesion in *METTL1*-depleted cells, firmly position DSG4 as the critical downstream effector underlying the phenotypic consequences of METTL1 deficiency. This suggests that the structural defects and cycling delays observed in our mouse models are largely driven by compromised desmosomal adhesion in the hair shaft. Therefore, our findings demonstrated the METTL1-HOXC13/FOXN1/DSG4 axis ensures the mechanical robustness of the hair follicle during its growth and regeneration.

### Limitation

A key limitation of our current model is the lack of the *in vivo* functional rescue experiments to determine whether restoring *HOXC13* or *DSG4* expression levels can ameliorate the hair follicle defects in *METTL1*‑deficient mice. Whether these molecular changes directly underlie the observed *in vivo* phenotype therefore remains to be further validated in future studies. While our data supports a model in which METTL1-mediated internal m^7^G modification of *HOXC13* mRNA contributes to its stability and translational efficiency, we cannot exclude the possibility that changes in tRNA m^7^G modification and/or tRNA abundance following *METTL1* depletion indirectly influence *HOXC13* expression or global translation. Furthermore, a deeper investigation into the transcriptional network downstream of HOXC13 is also warranted. We believe that these limitations do not detract from the main conclusions of the study but rather highlight important areas for future investigation.

In conclusion, our study reveals an essential role of METTL1-mediated internal m⁷G methylation of *HOXC13* mRNA in sustaining the hair follicle cycle. This work expands our understanding of how RNA modifications regulate hair follicle development and regeneration. Our findings further provide a mechanistic basis for exploring the contribution of RNA modification-dependent pathways to hair disorders, including thinning hair and alopecia.

## Materials and methods

The antibodies and sequences of the primers used in this study are listed in S1–S4 Tables.

### Ethics statement

All animal-related procedures were approved by the Animal Ethics Committee of Sichuan University (approval No.20220420002) and performed in compliance with the ARRIVE 2.0 guidelines.

### Animals

Animal housing was a specific pathogen-free (SPF) environment with sterile feed and a 12-hour light/dark cycle.

*K14-Cre* and *K14Cre-ER* C57BL6/J transgenic mice were kindly provided by Dr. Demeng Chen from Sun Yat-sen University (Guangzhou, China). *Mettl1*^*fl/+*^ C57BL6/J mice and *Mettl1KI*^*fl/+*^ were established by CRISPR-Cas9 technology in Beijing Biocytogen Biotechnology as reported previously [9,40]. We separately mated *Mettl1*^*fl/+*^ mice with *K14-Cre* and *K14-CreER* mice to obtain *K14*^*Cre*^*;Mettl1*^*fl/+*^ mice and *K14*^*CreER*^*;Mettl1*^*fl/+*^ mice, which were crossed with *Mettl1*^*fl/+*^ or *Mettl1*^*fl/fl*^ mice to obtain the final *K14*^*Cre*^*;Mettl1*^*fl/fl*^ mice and *K14*^*CreER*^*;Mettl1*^*fl/fl*^ mice. Using the same procedure, we obtained *K14*^*CreER*^*;Mettl1KI*^*fl/+*^ mice and their wild type littermates.

Our study incorporated sex as a biological variable by examining both male and female animals, and consistent phenotypes were observed for both.

### Histology and immunofluorescence staining

The dorsal skin slides were dissected from mice at different time points and then immersed in 4% PFA for over 24 hours at 4 °C. Then, the tissues were embedded in paraffin or optimum cutting temperature compound and sliced into slides ready for use. For immunohistochemistry and immunofluorescence, the slides were treated with 3% hydrogen peroxide (H_2_O_2_) or 0.3% TritonX-100 for 10–15 min after heat-induced epitope retrieval using EDTA. Then after blocked with 5% BSA, the slides were incubated with specific antibody overnight. Washed by PBS for 3 times, slides were incubated with the appropriate secondary antibody for 1 hour, followed by an AEC Staining Kit (Boster Biological Technology, USA) or 4,’6-diamidino-2-phenylindole (DAPI) according to the manufacturer’s instructions. As for TUNEL staining, the TUNEL Staining Kit (Promega, USA) was used as manufacturer commanded.

### Small interfering RNAs (siRNAs), single-guide RNA (sgRNAs) and lentiviral constructs

Small interfering RNAs (siRNAs) and single-guide RNAs (sgRNAs) targeting METTL1 were synthesized by Sangon Biotech (Shanghai, China). For *METTL1* depletion, HaCaT and HEK293T cells were transfected with siRNAs or sgRNAs using Lipofectamine 3000 (Invitrogen, USA) according to the manufacturer’s instructions. To generate single-cell–derived clones from mixed cell populations, limiting dilution was performed. For overexpression studies, a plasmid encoding catalytically inactive METTL1 (L160A/D163A) was constructed using the pLV3-CMV lentiviral vector. METTL1 was fused at its C-terminus with an S tag and an HA tag to facilitate protein detection.

### Lentiviral production and infection

Lentiviral particles were produced in HEK293T cells by co-transfecting the pLV3-CMV-METTL1 (L160A/D163A) plasmid or pLV3-CMV-DSG4/HOXC13 (human) -3 × FLAG-CopGFP-Puro plasmid with the packaging plasmids psPAX2 and pMD2.G using Lipofectamine 3000 and P3000 reagent (Invitrogen, USA). Viral supernatants were collected at 48 hours post-transfection, filtered through a 0.45 μm filter, and used to infect target cells in the presence of polybrene. Infected cells were selected with puromycin for analyzed as indicated.

### Cell adhesion assay and intercellular adhesion assay

For Cell counting kit-8 (CCK-8) assays, HaCaTs were seeded in 96-well plates and cultured for 12 hours. Then PBS was used to wash the unadhered cells for up to 3 times. HaCaTs were then treated with CCK-8 Reagent (Dojindo Laboratories, Japan) for 1 hour. And then measurements were then made with a spectrophotometer (Thermo Fisher Scientific, USA) at 450 nm.

For dispase-based dissociation assays, HaCaTs were seeded in 24-well plates and cultured until the cell density reaches 90%. After washed by PBS adequately, the HaCaTs were treated with dispase II solution (50mg/10ml in HBSS; Sigma-Aldrich D4693) for 15 min. Adding HBSS to stop dissociation, a constant mechanical shear stress was applied using an electrical pipette (Eppendorf, Hamburg, Germany), for 10 times each well [41]. The more fragments there are, the lower the adhesion between cells.

### Western blot, Northern blot and Northwestern blot

These experiments were performed as previously mentioned [42,43]. For Western blot, the total protein samples from cells were added and resolved in SDS–polyacrylamide gels, followed by transferred onto polyvinylidene fluoride membranes, which were then incubated with antibody against METTL1 (1:2000), DSG4 (1:1000), FOXN1 (1:1000), HOXC13 (1:1000), and α-TUBULIN (1:2000) at 4°C. The next day, the membranes were treated with the appropriate secondary antibody. For Northern blot and Northwestern blot, the total RNA samples were added and resolved in 15% Urea-PAGE gels, followed by transferred onto nylon membranes. Then the membranes were subjected to ultraviolet cross-linking. Then the membranes for Northern blot were incubated with the Digoxigenin-labeled U6 probe at 37°C overnight, followed by incubated with blocking buffer and horseradish peroxidase-conjugated antibody against Digoxigenin. The membranes for Northwestern blot were treated the same as Western blot. All the blots were imaged by the ECL system(Bio-rad, USA).

### Dual-luciferase reporter assay

The target *HOXC13* dual luciferase reporter plasmid synthesized by Jikai Gene (Shanghai, China) was transferred to *METTL1*-depletion 293Ts using Lipo3000. The cell sample preparation and assay procedure were both followed by the Dual-Luciferase Assay System Kit (Promega, USA).

### mRNA stability assay

WT and *METTL1*-KO 293Ts were seeded in 12-well plates. The total RNA was extracted after added actinomycin D (2 µg/ml) for 0, 0.5, 2, and 4 hours using trizol. The cDNA synthesis and qRT-PCR was performed using PrimeScript RT reagent Kit (Takara, Japan), ABI7500 real‐time PCR system (Applied Biosystems, USA) and SYBR Premix Ex Taq II (Takara, Japan). All the relative gene expression was calculated using the 2 − ΔΔCt method by normalizing to GAPDH.

### m^7^G-MeRIP assay

The enrichment of internal mRNA fragments with m^7^G modification were performed as previously mentioned [15,44]. The mRNA was extracted using Dynabeads mRNA DIRECT Purification Kit (61011, Invitrogen) and was fragmented into around 100 nt by RNA Fragmentation kit (AM8740, Invitrogen) as the manufacturer suggested, followed with the decapping procedure (Tobacco Decapping Plus 2, Enzymax) and purification. The 3’ de-phosphorylation and 5’-phosphorylation of these RNA fragments were performed using T4 Polynucleotide Kinase (EK0032, Thermo Fisher Scientific) following the manufacturer’s protocols. Then these repaired mRNA fragments (2 μg) were incubated with anti-m^7^G antibody (1:75) or IgG antibody (1:75) with 5% SUPERase-In RNase inhibitor (Thermo Fisher Scientific) for 3 hours at 4 °C. Next, after washed and resuspended, an appropriate amount Dynabeads Protein G resins (Thermo Fisher Scientific) were added into above-mentioned mixture and incubated for 2 hours at 4 °C. After washed thoroughly, the RNA was eluted using Proteinase K (Thermo Fisher Scientific) and recovered with RNA Clean & Concentrator (Zymo Research). The cDNA synthesis and qRT-PCR was performed as above-mentioned.

### RNA-sequencing

A total of 4 μg RNA per sample was isolated from dorsal skin epidermis at P5. Sequencing libraries were prepared by Novogene Co., Ltd using NEBNext Ultra RNA Library Prep Kit for Illumina (NEB, USA), with an index code added to the attribute sequence of each sample. HISAT2 v2.0.5 was used for constructing the reference genome index and aligning the paired-end clean reads with the reference genome. Read counts were determined by featureCounts v1.5.0-p3. The expression level of genes was estimated by fragments per kilobase million mapped reads (FPKM). The differential expression genes (DEGs) were identified with q ≤ 0.05 and log2_ratio| ≥ 1. For gene ontology (GO) and KEGG enrichment analysis, DEGs were analyzed in the Database for Annotation, Visualization, and Integrated Discovery. GO and KEGG enrichment analyses were conducted using the hypergeometric test. GO and KEGG terms were considered significantly enriched with q  <  0.05.

### Quantification and statistical analysis

GraphPad Prism 9 (GraphPad Software, USA) and SPSS were used for statistical analyses in this study. Two groups were compared using an unpaired, two-tailed t-test, while one-way ANOVA followed by Tukey’s post-hoc test was used for the comparison of multiple groups. P values <  0.05 were considered significant.

## Supporting information

S1 Fig Representative images of barrier function assay of *K14*^*Cre*^*;Mettl1*^*fl/fl*^ and *K14*^*Cre*^*;Mettl1*^*fl/+*^ mice. (TIFF)

S2 Fig Representative TUNEL staining of dorsal skin of *K14*^*Cre*^*;Mettl1*^*fl/fl*^ and *K14*^*Cre*^*;Mettl1*^*fl/+*^ mice. Scale bar, 50 μm.
(TIFF)

S3 Fig Conditional knockout of *Mettl1* delayed hair regeneration. A. Representative immunohistochemical staining images of the HFs from *K14*^*CreER*^*;Mettl1*^*fl/fl*^ mice and *K14*^*CreER*^*;Mettl1*^*fl/+*^ mice. Scale bar, 100μm. B. Representative and detailed images of hair regeneration from *K14*^*CreER*^*;Mettl1*^*fl/fl*^ mice and *K14*^*CreER*^*;Mettl1*^*fl/+*^ mice after tamoxifen induction and hair-shaving treatment (male).
(TIFF)

S4 Fig Depletion of *Mettl1* impairs the intercellular adhesion of keratinocytes. A. Quantitative analyses of cell adhesion experiment using CCK-8 assay in *siCTRL* and *siMETTL1* HaCaTs (n = 4). B. Quantitative analyses of Western blot of METTL1, HOXC13 and DSG4 (n = 3).
(TIFF)

S5 Fig METTL1 mediated internal mRNA m^7^G modification of *HOXC13.* A. Bar plot of dual-luciferase assay in *siCTRL* and *siMETTL1* 293Ts (n = 3). B. Schematics of the enzymatic active site of METTL1. C. Quantitative analyses of Western blot of METTL1 (n = 3). D. Quantitative analyses of Northern blot and Northwestern blot of m^7^G signals (n = 3).E. tRNA m^7^G decoding codon abundance between the background genes and *HOX13* families.
(TIFF)

S1 Table List of primary and secondary antibodies used. (XLSX)

S2 Table List of primer sequences for genotyping. (XLSX)

S3 Table List of primer sequences for qRT-PCR. (XLSX)

S4 Table List of sequences of siRNA and sgRNA. (XLSX)

S5 Table The detail information of the internal m^7^G modification site on HOXC13 mRNA using public data (site 1). (XLSX)

S1 Raw Gel Raw image. (PDF)

S1 Data Raw Data. (XLSX)
