# Supplementary material for: METTL1-mediated m7G modification regulates hair follicle cycle via the HOXC13/FOXN1/DSG4 axis
Source: PLoS Genet. 2026 Sep 22;22(9):e1012307. doi: 10.1371/journal.pgen.1012307 (PMC13596829; doi:10.1371/journal.pgen.1012307)

F5C

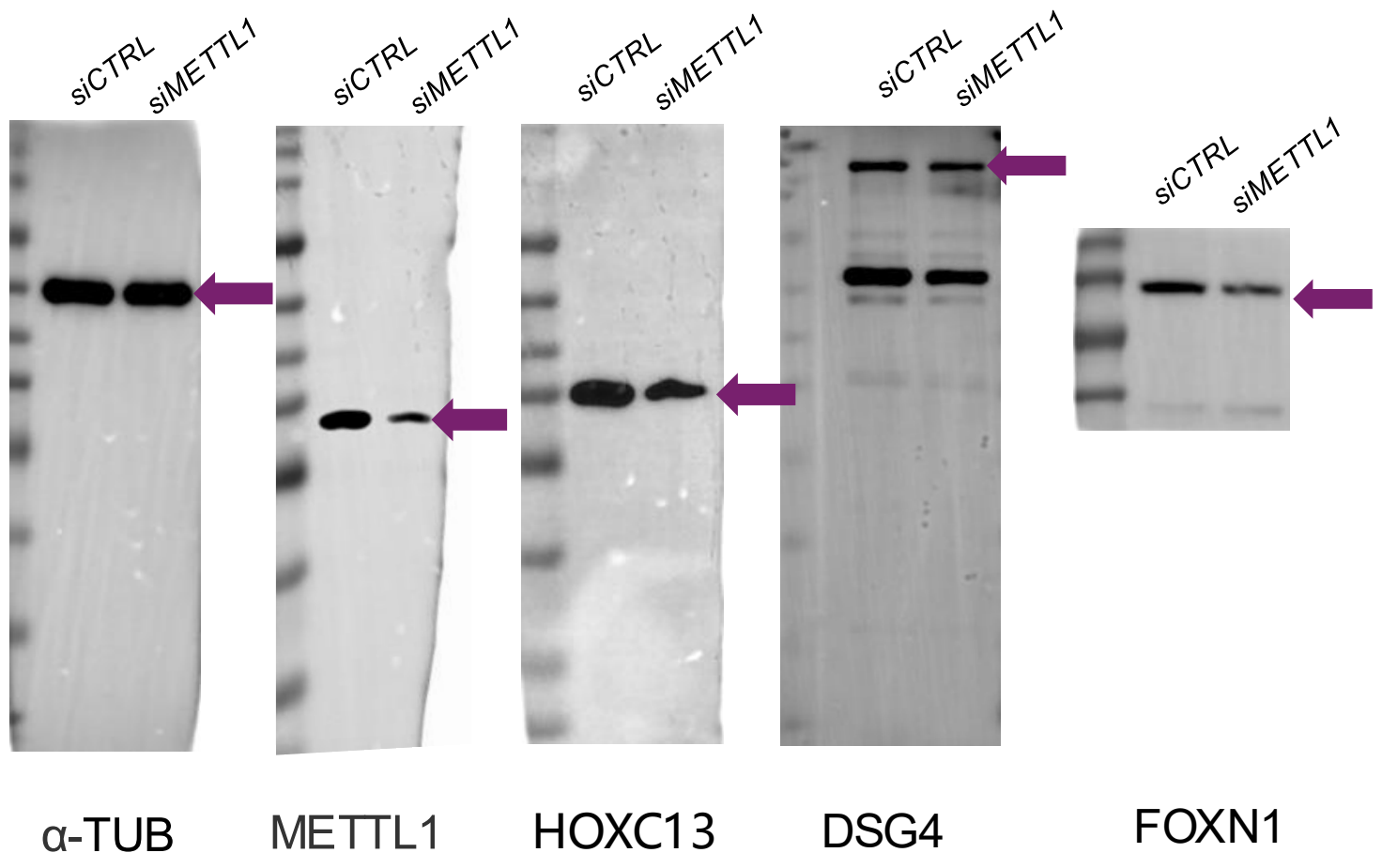

F5I

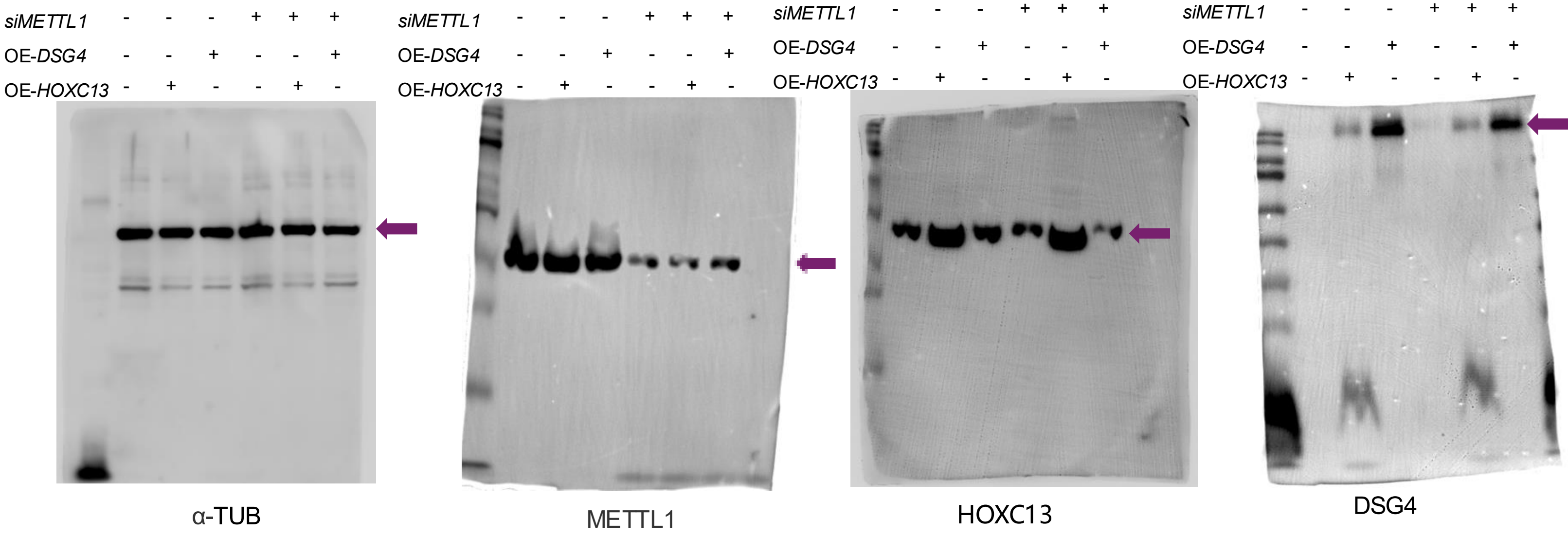

F6A

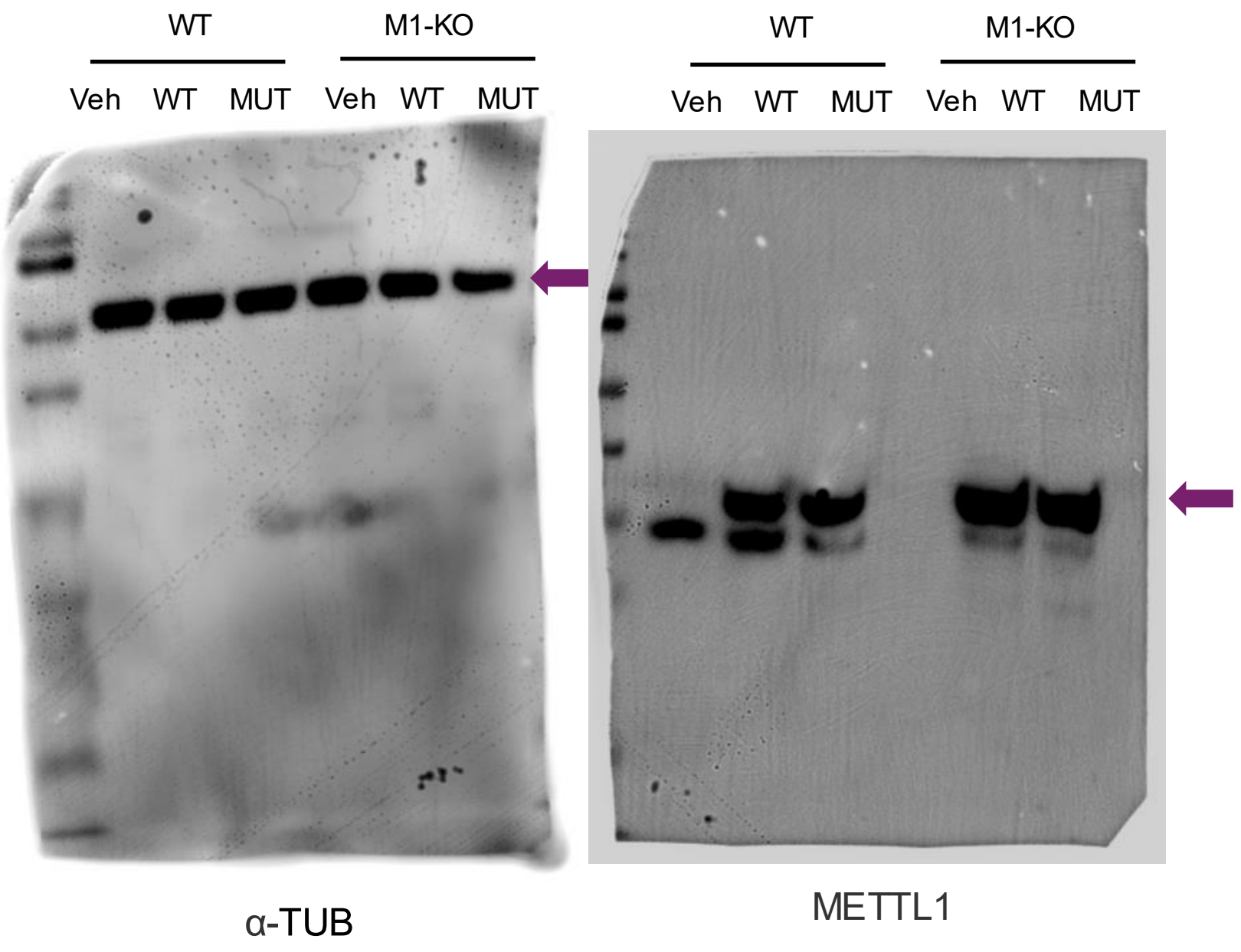

F6B

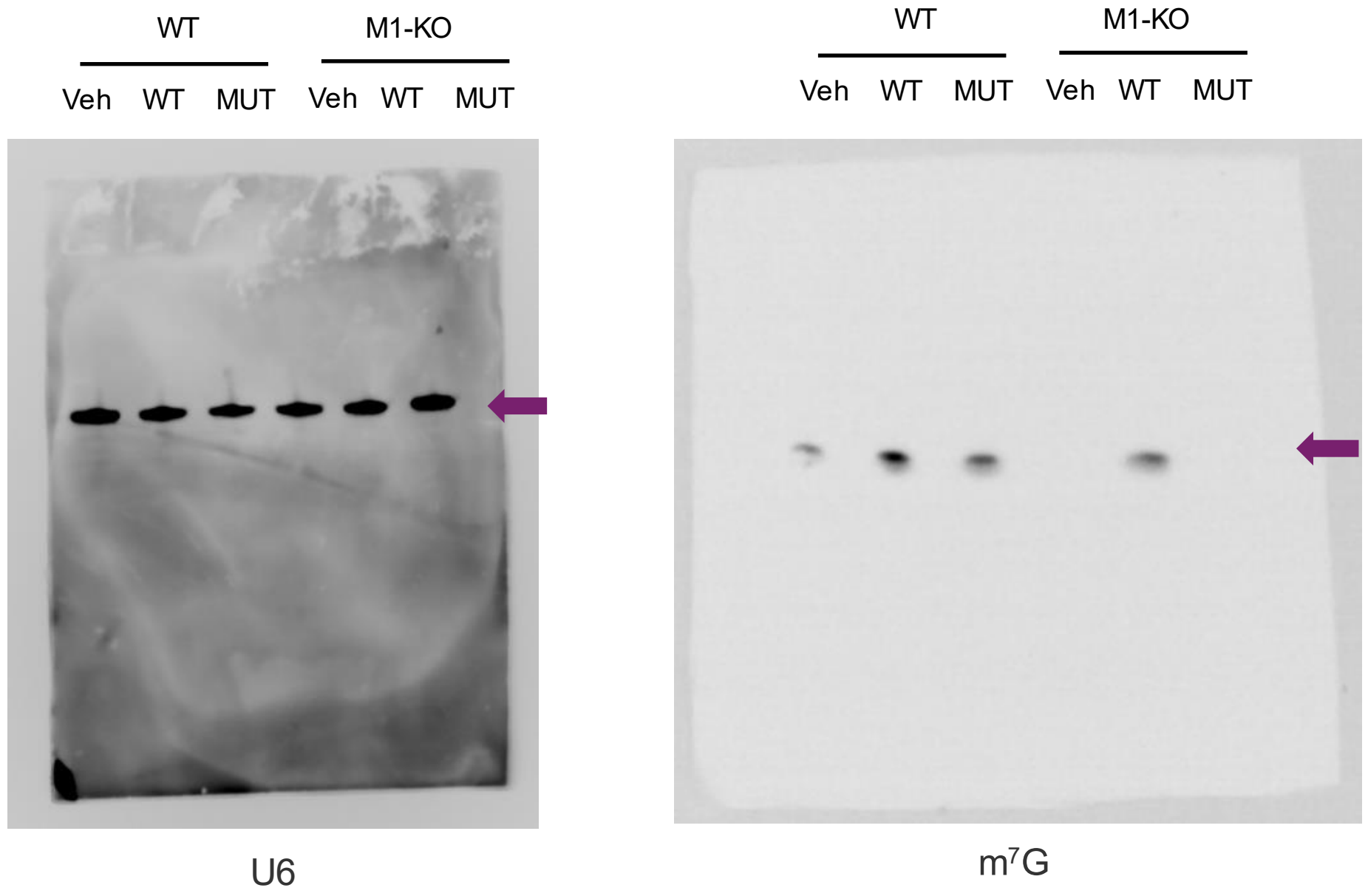

Supplement: Supplementary file 1 [file pgen.1012307.s011.pdf]
